# How does pre-dialysis education need to change? Findings from a qualitative study with staff and patients

**DOI:** 10.1186/s12882-017-0751-y

**Published:** 2017-11-23

**Authors:** Gill Combes, Kim Sein, Kerry Allen

**Affiliations:** 1 Institute of Applied Health Research, University of Birmingham, Birmingham, B15 2TT UK; 2 Hull York Medical School, University of Hull, Hull, HU6 7RX UK; 3 Health Services Management Centre, University of Birmingham, Birmingham, B15 2TT UK

**Keywords:** Pre-dialysis education, Patient decision-making, Renal replacement therapy, Emotional support, Counselling, Patient choice

## Abstract

**Background:**

Pre-dialysis education (PDE) is provided to thousands of patients every year, helping them decide which renal replacement therapy (RRT) to choose. However, its effectiveness is largely unknown, with relatively little previous research into patients’ views about PDE, and no research into staff views. This study reports findings relevant to PDE from a larger mixed methods study, providing insights into what staff and patients think needs to improve.

**Methods:**

Semi-structured interviews in four hospitals with 96 clinical and managerial staff and 93 dialysis patients, exploring experiences of and views about PDE, and analysed using thematic framework analysis.

**Results:**

Most patients found PDE helpful and staff valued its role in supporting patient decision-making. However, patients wanted to see teaching methods and materials improve and biases eliminated. Staff were less aware than patients of how informal staff-patient conversations can influence patients’ treatment decision-making. Many staff felt ill equipped to talk about all treatment options in a balanced and unbiased way. Patient decision-making was found to be complex and patients’ abilities to make treatment decisions were adversely affected in the pre-dialysis period by emotional distress.

**Conclusions:**

Suggested improvements to teaching methods and educational materials are in line with previous studies and current clinical guidelines. All staff, irrespective of their role, need to be trained about all treatment options so that informal conversations with patients are not biased. The study argues for a more individualised approach to PDE which is more like counselling than education and would demand a higher level of skill and training for specialist PDE staff. The study concludes that even if these improvements are made to PDE, not all patients will benefit, because some find decision-making in the pre-dialysis period too complex or are unable to engage with education due to illness or emotional distress. It is therefore recommended that pre-dialysis treatment decisions are temporary, and that PDE is replaced with on-going RRT education which provides opportunities for personalised education and on-going review of patients’ treatment choices. Emotional support to help overcome the distress of the transition to end-stage renal disease will also be essential to ensure all patients can benefit from RRT education.

## Background

Every year in the United Kingdom, 7000 patients with end-stage chronic kidney disease (CKD stage 5) start a renal replacement therapy (RRT) for the first time [1]. There are five main treatment options open to these patients: transplantation; haemodialysis in a nurse-led unit/hospital; haemodialysis at home; peritoneal dialysis; and conservative care. Each option has different clinical advantages and disadvantages, and different impacts on patients’ lives. This makes the selection of RRT quite complex. In line with national policy, renal services promote patient choice of treatment, within a framework of clinically feasible options. Most patients therefore undertake pre-dialysis education (PDE) over a number of months prior to starting RRT, which is designed to help them make a treatment decision. Although practice varies, PDE usually includes one or more one-to-one sessions with a specialist nurse; a group meeting with talks from clinical staff and patients already on RRT; and written and audio-visual materials to take home.

Although the importance of PDE was highlighted in 2010 by the European Renal Best Practice Advisory Board [2], research into its effectiveness is still in its infancy. Studies consistently report that one-third or more of patients do not recall receiving information about treatment options [3–5]. Patient dissatisfaction with various aspects of PDE was found in two recent large studies, one in the US [6] and the second in 36 European countries [4]: some patients felt not all treatment options were presented equally [4, 6]; others could not recall being told about options other than their current treatment [4]. Satisfaction with education about transplantation and in-centre haemodialysis is often higher than for PD and home haemodialysis [4, 6], although a more recent Australian study found no significant differences in knowledge between patients on different types of treatment [7].

Smaller studies suggest that PDE is sub-optimal because: patients lack information or feel choices are limited [8]; education may be provided too late, when patients are too ill to make decisions [8]; individual healthcare professionals have a bias towards/against certain treatments [7, 9]; patient information is too complex or hard to understand [8, 10, 11]; information may not stress that patients have choices [11] or may not consider sufficiently patients’ preferences and lifestyles [12]; and patients may not be as involved in treatment decision-making as they would prefer [13, 14].

In the absence of a high-quality evidence base, national guidelines have been developed using consensus-building techniques. In 2014, UK renal experts published three patient education standards covering: the importance of education in supporting patient choice; the need to tailor education to individual needs; and the continuation of education into the RRT treatment phase [15]. In 2015, European experts published quality standards, making recommendations about the content, timing, delivery and evaluation of PDE [16]. To our knowledge, there have been no trials of enhanced PDE designed to address these shortcomings, although there have been two recent trials of RRT decision-aids [7, 17].

In 2011–12, we undertook a 4-site mixed methods study looking at the barriers and success factors for home dialysis treatment and the influence of a target on uptake rates [18]. Since this study found that PDE was one of three main barriers to increasing the uptake of home dialysis, we subsequently decided to report the findings about PDE in more detail. Given that the original study set out to explore home dialysis, it is possible that this could have skewed the data and findings. However, we consider that the data we present are highly likely to be relevant to all dialysis patients because: patients were asked about their treatment pathways and how choices had been made *in general*, rather than specifically related to home dialysis; and it was only at the very end of the patient interviews, after talking about PDE, that views about home dialysis were explored. In this article, we report a complete analysis of data related to PDE from staff and patients from the main study, exploring the following question: how effective is PDE from the perspective of patients and staff?

## Methods

The main study used mixed methods to look at quantitative changes in home dialysis uptake rates and qualitative case studies to explore barriers and success factors for home dialysis. The setting was four hospital renal units, selected from seven West Midlands units to achieve a demographic and rural/urban mix. Semi-structured one-to-one interviews were undertaken with dialysis patients and clinical and managerial staff. An intellectual framework for the design and analysis of qualitative interviews, which has been reported in detail elsewhere [18], was derived by mapping systematic review evidence of potential success factors onto an established theoretical model for health system change, and cross-checking this for relevance against renal service guidance. The interview topic guides consisted of a small number of semi-structured open-ended questions designed to prompt the sharing of experiences and views. For patients, the topic guide covered: how patients came to be on dialysis; experiences of pre-dialysis and dialysis pathways; and suggestions for improvement. For staff, the topic guide covered: current practice, using the last 2–3 patients as exemplars; how well the pre-dialysis and dialysis pathways work; how the team had been working to increase the uptake of home dialysis; and suggestions for improvement. No direct questions about PDE were asked in either staff or patient interviews. If patients/staff did not spontaneously talk about the pre-dialysis period, they were prompted with an open-ended question about how treatment decisions were made.

The patient population was dialysis patients aged 18+ starting their current treatment within 12 months, excluding patients scheduled for surgery within 3 months as they were unlikely to be available or unfit to be interviewed. Purposive sampling by age, sex, ethnic group and treatment type was used to achieve maximum diversity. Potential patient participants were approached by phone by renal secretaries, who sent out study information to interested patients who were subsequently contacted by the research team. Staff participants were encouraged to take part and provided with study information via e-mail from the renal clinical lead, with renal secretaries then scheduling interviews. Semi-structured qualitative telephone interviews were undertaken with 20–25 patients per site (November 2011–March 2012) until saturation was achieved. The staff population was clinical staff working with CKD stage 5 patients and managerial staff. Semi-structured qualitative face-to-face interviews were undertaken on-site with 20–30 staff per site (Table 1) (September 2011–April 2012) until saturation was achieved. Interviews lasted for 30–60 min and were undertaken in private with only the interviewer and interviewee present. Brief field notes were made, as appropriate, after each interview. The interviews were shared equally between GC, KA and KS who were all experienced qualitative health service researchers, employed by the University of Birmingham. KS is a specialist in qualitative methods. This information was provided to participants via the Participant Information sheet. GC and KA are female and have Ph.Ds. KS is male and has an M.Sc. None of the research team: were clinically qualified/experienced; had any prior or current relationships with the four renal teams or NHS Trusts taking part in the research; had not undertaken previous research with end-stage renal patients/staff; and did not have personal experience or a particular personal interest in the research topic.

**Table 1:** Roles of staff interviewed

Staff job role	Hospitals					
	1	2	3	4	Total	Total %
Renal consultant lead	1	1	1	1	4	
Renal consultant	8	6	3	2	19	
Clinical specialist	–	–	–	1	1	
Specialist registrar	2	2	1	–	5	
Sub-total doctors	11	9	5	4	29	30%
Acute ward nurse manager	2	1	1	1	5	
Dialysis unit nurse manager	3	3	4	3	13	
Lead renal nurse/renal matron	1	–	–	1	2	
Pre-dialysis nurse/sister	1	1	3	1	6	
PD nurse/sister	2	–	–	2	4	
Home therapy nurse	–	4	3	–	7	
Home haemodialysis nurse/sister	2	–	–	2	4	
Sub-total nurses	11	9	11	10	41	43%
Home therapy support worker	–	1	–	–	1	
Renal technician	1	1	1	1	4	
Psychologist	–	–	–	–	0	
Dietitian	1	1	–	1	3	
Consultant vascular surgeon	–	1	1	–	2	
Renal social worker/assistant	1	–	–	1	2	
Renal business manager	1	–	1	1	3	
Sub-total other renal staff	4	4	3	4	15	16%
Hospital general managers	2	1	–	1	4	
Hospital clinical/medical director	1	2	1	1	5	
Hospital finance manager	1	–	–	1	2	
Sub-total hospital managers	4	3	1	3	11	11%
TOTAL	30	25	20	21	96	
Kidney Patients Association chair	1	–	–	1	2	
No. interviews declined	3	0	7	0	10	

Tables 2 and 3 summarise the characteristics of eligible and interviewed patients. The eligibility criteria were amended during fieldwork in site 1 to include patients starting treatment within the last 24 months, rather than 12 months, as there were few eligible patients in some sampling categories. No effects were observed from this change, particularly on patients’ abilities to recall their treatment experiences. Of the 618 patients who had started their current dialysis treatment within the last 24 months, 101 patients (16%) were invited to interview, 8 refused and 93 were interviewed (21–25 per site). Of the 106 staff invited to interview, 10 refused and 96 were interviewed (20–30 per site). Table 1 details the roles of staff interviewed. There were no withdrawals of patients or staff from the study. All interviews were audio-recorded and were transcribed verbatim by a specialist transcription team. Transcripts were checked by researchers but not participants. The written and audio-visual PDE materials used in each site were also reviewed.

**Table 2:** Patient sampling

Patient sample	Hospitals				
	1	2	3	4	Total
Eligible	205	152	129	132	618
Refusals	–	5	3	0	8
Interviewed	23	25	21	24	93
% eligible patients interviewed	11%	16%	16%	18%	15%

**Table 3:** Patient characteristics

Patient characteristics	Hospitals						No. eligible patients	% eligible patients interviewed
	1	2	3	4	Total	%		
Treatment type								
PD	10	11	11	8	40	43	181	22%
Home haemodialysis	4	7	1	6	18	19	28	64%
In-centre haemodialysis	9	7	9	10	35	38	409	9%
Sex^a^								
Male	14	18	12	11	55	59	359	15%
Female	9	7	9	13	38	41	230	17%
Age group								
18–39	5	5	3	5	18	19	67	27%
40–64	13	8	8	9	38	41	223	17%
65+	5	12	10	10	37	40	328	11%
Ethnic group^a^								
White	13	25	15	23	76	82	509	15%
Indian	6	0	2	1	9	10	52	17%
Pakistani	2	0	0	0	2	2	23	9%
African Caribbean	2	0	4	0	6	6	33	18%

^a^Missing data: sex not recorded for 29 eligible patients not included in the study; ethnic group not recorded for 10 eligible patients not included in the study

### Analysis

Data were analysed using a form of thematic analysis [19], the framework method [20], which has been shown to be useful in conducting healthcare research with a multi-disciplinary team of researchers. This allowed the development of themes to be derived entirely from the raw data to provide rich descriptions of how patients experienced PDE and what might need to improve. Researchers familiarized themselves with the audio-recordings and transcripts, and analysed a small number of entire transcripts line by line to generate initial codes, which were then compared, refined and agreed on as a team. An analytic framework was developed from the initial group of transcripts and then refined as the full set of transcripts was coded onto a spreadsheet using a matrix of codes and cases. Coding was shared equally between GC, KA and KS with 10% of transcripts coded by two researchers and discrepancies resolved at team meetings. The resulting themes were refined through team discussion. Separate analysis of staff and patient transcripts at each site were then triangulated. Discussion of findings with clinical staff at site-specific feedback meetings led to further refinement, followed by triangulation and synthesis across sites to identify overall study findings. Research team meetings provided the forum for discussing reflexivity and considering how to minimise the influence of individual researchers on the research.

For the analysis presented in this article, transcripts were subsequently re-read, checking that a complete data set on PDE had been extracted: to include all direct mentions of PDE and treatment decision-making, and more general comments about the pre-dialysis period; and to exclude any data linked to or arising from prompts about home dialysis. No data were identified for exclusion as a result of this checking. The themes identified for PDE were not specified in advance, but were derived entirely from the data.

## Results

Formal PDE in all four sites included: one or more one-to-one sessions with a specialist nurse; a group information session, including talks from patients on RRT; and written materials/DVDs which patients took home. In several sites, specialist nurses undertook home visits where they discussed treatment options with patients. Doctors also discussed treatment options with patients during out-patient appointments. Most staff made favourable comments about PDE and valued the role of specialist nursing staff in educating and supporting patients’ treatment decisions. Most patients recalled taking up part or all of the formal PDE on offer and reported finding it helpful overall. Three themes related to improving PDE were identified (Table 4): sub-optimal education; different perspectives between patients and staff; and the influence of patient experience. These themes are explored, using quotes from patients and staff to illustrate the main points, with diverse cases noted within themes.

**Table 4:** Themes and sub-themes

Themes	Sub-themes
Sub-optimal education	Restricted range of teaching methods and materials Bias in the presentation of information and treatment options
Different perspectives between patients and staff	The importance of informal education Approaches to treatment decision-making
The Influence of patient experience	How other patients can influence decision-making The impact of distress

### Sub-optimal education

#### Restricted range of teaching materials and methods

Although some patients were critical of the volume and types of information about treatment options they had been given by staff and the methods that were used in PDE, there were no concerns about these issues from staff. Although patients need information to make treatment decisions, some felt they had been unable to use it because the volume and complexity of information meant they were unable to understand or assimilate it:

*“You get all this information and that’s the information overload bit…. But you’re not really taking it in.” Patient 15, site 4 (PD, female, white, aged 18–39)*.

From the staff perspective, the deliberate reliance on written materials was designed so that patients had information to take home and consider over time. However, it seemed that some patients were unable to take advantage of this positive intention. This was particularly the case for one patient, whose dyslexia had not been catered for, leaving her with written educational materials that demanded a high level of reading skills. Although this was only one patient, it is possible that other patients’ dissatisfaction with the written materials could be explained partly by difficulties with reading. Additional limitations in the range of materials were noted: a lack of information for patients whose first language was not English; a paucity of computer-based materials; and low confidence amongst staff in signposting patients to reputable and relevant websites.

Another perspective on teaching materials came from patients who thought that they were not ‘real’ enough, and that this explained why they had struggled to apply the information to their own lives. Seeing different treatments being undertaken by real patients, being able to see and touch the equipment, and being able to talk to patients already on treatment about what it felt like and how they experienced it, were all suggested as ways of improving the education:

*“And actually see something, you know, like see a haemodialysis machine, a PD [machine], rather than just… its different seeing it on the page than actually seeing it in real life.” Patient 5, site 1 (PS, female, white, aged 40–64).*

*“I was given lots of stuff [information] but really I needed to go out and see a couple of people [having treatment]… to see how it suited them…it doesn’t really matter what the nurses say, its how it affects people really, that’s why I wanted to go and see them.” Patient 9, site 4 (HD, male, white, aged 40–64).*

This suggests that patients would benefit from the use of a wider range of teaching methods, including interactive methods.

#### Bias in the presentation of information and treatment options

Whilst some patients thought that all treatment options were presented fairly and with equal emphasis, others felt not all options had been presented to them and that they had only found out about viable alternatives once they were on dialysis.

*“Erm, yeah there was no preference from the hospital side I don’t think I was never pressured to do either [treatment].” Patient 4, site 3 (PD, male, white, aged 40–64).*

*“She [the nurse] didn’t really give me any choice, no. She just recommended dialysis [HD] and that was it.” Patient 18, site 3 (HD, male, white, 65+).*

Following discussion of this issue with clinicians, analysis showed that these patients were evenly distributed across treatment types and did not include a disproportionate number of acute kidney injury patients, for whom choices could have been restricted.

There was also a view from patients that staff were overly positive about dialysis, which had not prepared patients well for side-effects or the impacts of treatment on day-to-day life:

*“They [nurses] only look on the good side of everything.” Patient 17, site 1 (PD, female, white, aged 40–64).*

*“…more information on the like, not so positive side of it, you know, because there is a good side of being on dialysis and there is a bad side.” Patient 16, site 1 (PD, male, white, aged 40–64).*

Some of these patients thought that opportunities to talk to patients already on treatment might have helped to give them a more balanced view of what life on dialysis might be like.

Staff were also aware of the potential for bias, and that their position as a trusted health professional could potentially lead to them having undue influence over patients. However, all staff groups thought that the first conversation that doctors have with patients about treatment options is crucial in influencing treatment choice. If doctors appear to favour a particular treatment, no amount of PDE can counteract the influence this initial conversation has over patients’ eventual choice:

*“I do think patients do get swayed, particularly by consultants, because they [patients] think they [doctors] know best. I think it’s the initial conversation that they have, you know, which I can only presume will be a consultant initial conversation...but if they’ve had that underpinning by the consultant first, it’s then very difficult [to influence them otherwise].” Renal ward sister, site 4.*

### Different perspectives between patients and staff

#### The importance of informal education

Staff tended to equate PDE with the work undertaken by specialist pre-dialysis nurses, whilst recognising that patients’ conversations with doctors also influence treatment decision-making. These opportunities for PDE were similarly recognised and valued by patients. However, patients were equally gathering information and views about treatment options through their informal contacts with staff, for example, chatting to staff whilst waiting for appointments or during in-patient stays. This emerged as significant because some of these patients talked about these groups of non-specialist staff as being less knowledgeable about the full range of treatment options and often unable to answer patients’ questions or signpost them to appropriate sources of information and advice. Only a handful of staff thought likewise:

*“I think a lot of effective patient education is delivered through everyday conversation and chat.” Pre-dialysis nurse, site 4.*

This same member of staff recognised that this means that all staff, wherever they work, should be equipped to deal with casual questions from patients:

*“You know somebody [a patient] might ask a question. Well if staff haven’t got the knowledge… then that conversation isn’t going to go any further.” Pre-dialysis nurse, site 4.*

However, nurses working on the wards and in haemodialysis units reflected that they felt ill-equipped to talk with patients about all treatment options due to lack of training or lacked experience of the full range of treatment options. For some, this seemed to be explained by the trend towards specialisation, and to remedy this, several sites had recently introduced staff rotations:

*“They’ve tended to be employed and they’ve stuck where they are… so when you get newly qualifieds just going straight into haemo. and not even done any ward work, they can’t see the whole picture then and can’t advise patients on what its like to go on PD because they’ve not seen it.” Senior nurse, site 2.*

*“The staff will be starting to rotate… the benefit is that you end up with a renal nurse who knows all about everything… who even if they decided to work in haemo. permanently, can at least talk to the patient ‘ well this is what PD is about and this is what transplantation is about’.” Home therapies nurse, site 2.*

It was also apparent that some patients continued to consider treatment options well after they had started dialysis, and carried on gathering information and views about treatment options, some with a view to switching treatment. This highlighted the importance of *all* staff, irrespective of their role, being able to present all options neutrally and answer basic questions about all types of treatment.

#### Approaches to treatment decision-making

A second issue, about which staff and patients appeared to have very different perspectives, was in how they viewed treatment decision-making. Nearly all staff described a rational fact-based approach to treatment decision-making, where patients would use detailed information to weigh up treatment options:

*“You must make sure as a doctor or a service that you’ve given the patients all the information, given them enough time to come to terms with what’s required, help them to, support them with their choices.” Consultant, site 2.*

*“We just have to tell about the treatments and it’s up to them [patients] to choose….You tell them about all other forms of treatment as well so then it’s up to the patient to make a choice.” Consultant, site 4.*

This rational decision-making approach was also reflected in the written PDE materials provided in all sites. It contrasted with the patients, who mostly talked about a more personalised approach of thinking about their own lives and how different treatment options might work for them. For some, there appeared to be one main reason for their choice of treatment, which was often non-clinical and highly relevant to each individual’s life:

*“I made up my mind on doing the PD one because it still allowed me to carry on working.” Patient 7, site 1 (PD, male, Indian, aged 18–39).*

*“If I’ve got the choice I think I would still prefer hospital for the simple reason that there’s qualified staff on the scene. If anything goes wrong you’ve got qualified staff there and with you.” Patient 18, site 3 (HD, male, white, 65+).*

For others, decisions seemed to be influenced primarily by a fear of the unknown or anxiety about making a decision, rather than the rational processes described by staff. Only one member of staff seemed to fully appreciate the difficulty patients faced when making a choice:

*“Asking people to make a choice I think is a mixed blessing, ‘cos I think it causes extra anxiety and stress to patients when they’ve got to be making the decision about what to do. And I think some people can’t, they just don’t feel they can make the decision.” Pre-dialysis nurse, site 4.*

### The influence of patient experience

#### How other patients can influence decision-making

The influence of other patients on decision-making had several aspects. Firstly, some patients valued having opportunities to talk to other patients, particularly those who were already on dialysis, because they were able to portray what treatment is really like:

*“Speaking directly to someone who has had it [dialysis], so you’re getting all the unfiltered information...it was useful to be able to speak to a person who had gone through that to give us, you know, warts and all what’s going to happen, so that was good.” Patient 15, site 4 (PD, female, white, aged 18–39).*

*“I mean, patients can be talked to by professionals, nurses or doctors and what have you, but I think they’ve got to, you know, another patient, a fellow patient just has that more credibility.” Patient 2, site 2 (HHD, male, white, aged 65+).*

Secondly, some patients thought this helped to balance any biases from staff:

*“I think the nurses, although they’re very good there, they kind of just look at it from one side don’t they? If you talk to a patient he’s going to tell you how it’s sort of happened to him.” Patient 4, site 1 (HD, Male, Indian, aged 40–64).*

Some staff also recognised that pre-dialysis patients can find it very helpful to talk to patients already on RRT:

*“I suppose successfully, the patients the way they take the information, often the most potent thing is speaking to the patient next door or in the waiting room and they say ‘oh you can’t have that one I was awful on that’.” Consultant, site 2.*

However, other staff were more cautious and actively discouraged patient contact, because some patients may have atypical experiences or be biased against certain treatments:

*“Patients are swayed far more by what they hear from other patients than all the information we give them.” PD sister, site 4.*

Interestingly, none of the patients had been offered pre-dialysis opportunities to talk to other patients, although this had recently been introduced in one site, and a second site would put patients in touch with each other on request.

#### The impact of distress

The impact of distress on decision-making emerged as a strong theme across all patient groups and sites. Patients described at length, the traumatic and frightening nature of the transition to end-stage renal failure. It seemed likely that this might help to explain why so many patients said they found treatment decision-making very difficult, including those patients who had known for years they would need RRT and who might therefore be expected to be well prepared for treatment decision-making:

*“…they were explaining it to me but it just didn’t go through me head that I was going to get ill, like. I mean they were very, very nice but I was just too scared [to make a choice].” Patient 4, site 2 (HD, female, Indian, 40–64).*

Some patients were quite critical of the staffs’ focus on the factual and clinical aspects of treatment:

*“So they focus totally on the practical side of things….You’re going to die if you don’t do it [treatment]. It’s all very black and white, all very aggressive and you know perhaps that works for some people, it certainly doesn’t work for me….. [There’s] a huge mental side to it, well I don’t know what you’d call it, a psychological element they probably don’t quite press.” Patient 4, site 3 (PD, male, white, 40–64).*

However, very few staff appeared to appreciate the potential adverse impact of psychological distress on patients’ ability to make treatment decisions. Just three staff raised this issue in their interviews:

*“So quite often people are shocked, you know, they just kind of don’t know what to think really about anything, and even when they, even if they’ve had all the information, they start with us, they still need a lot of support, to kind of make the right choices really…. I kind of equate it to like the grieving really, they’ve lost their kidneys and its almost like a death for them… they kind of go through all those emotions that come with bereavement really.” Dialysis unit nurse manager, site 4.*

In contrast, one-third of the patients talked about the distress of going onto end-stage treatments, and for some, they had only become open to some treatment options once they had started on dialysis. Although some staff were aware of this, once patients had started on dialysis there were no additional routine education opportunities for patients in any of the sites, nor routine reviews of treatment choice, which could support patients to revisit their treatment choice:

*“People might start on one treatment and then six months down the line feel very differently… the education is, should be on-going, rather than you have it before you start and then you never have any more. …people do change their minds and gain confidence as they get used to a situation…” Pre-dialysis nurse, site 4.*

## Discussion

Although the study found that patients’ and staffs’ views about PDE were largely favourable, a number of suggestions for improvement and optimisation emerged. The literature supports the findings that some patients thought teaching materials and the way they were used could be biased [4, 7, 9, 12, 14, 19, 21], and that patients wanted a wider range of teaching methods to be used, particularly active learning methods [7, 22, 23] and seeing dialysis treatments in action [16]. The diversity in patients’ preferences for different teaching methods suggests it would be appropriate for a patient’s preferred learning style to be assessed ahead of starting PDE in line with the principles of adult learning [24].

As in other studies [25], patients were provided with lots of information, and some complained of information overload. They wanted less detailed factual information with more time spent on helping patients to apply information to their own lives, which suggests that PDE may need to be re-balanced away from a reliance on information-giving. Recent initiatives to develop and trial decision support tools [7, 17, 26] may go some way to helping with this. Likewise, opportunities for patients to talk to other patients already on RRT, could help them to envisage what life on dialysis is really like, as noted in previous studies [8, 27], and help to counter the perception that staff may be biased or overly positive about treatments. However, this would need to be implemented with care, given evidence that patients’ stories can bias other patients’ treatment choices, irrespective of clinical advice [28]. Whilst some of these improvements to PDE could be relatively easy to implement, the study identified two additional themes which potentially have more fundamental implications for PDE: differences in perspective between staff and patients; and the influence of patient experience.

Several important differences in perspective emerged from the data. Our study suggests that staff and patients may not conceptualise PDE in the same way, with staff focussing on formal PDE sessions and discussions during out-patient appointments, whilst patients appear to place additional value on more informal education, arising from conversations with staff and other patients. However, for this to contribute positively to patients’ treatment decision-making, staff who are not PDE specialists, from across the spectrum of renal services, would need to be informed enough to chat with patients about the full range of treatment options. This was not the case in this study. The small amount of relevant literature suggests this may be hard to achieve, as one study has found that renal nurses’ attitudes to RRT options are strongly associated with their own area of expertise and experience [9], whilst a second study has recommended that all staff who come into contact with patients need experience of all treatment types in order to talk confidently with patients [29].

The second notable difference in perspective between staff and patients was how treatment decision-making was conceptualised. Whilst staff thought patients should or do make decisions using a rational fact-based approach, patients mostly described a process of thinking about the possible impact of dialysis on everyday life and giving one highly individual reason for choosing their treatment. Although some previous studies have stressed the importance of renal patients using information to weigh options [7], many other studies suggest that RRT treatment decision-making is not as simple as this, and that patients make decisions which accord with the context of their lives, values and identity [25, 27, 29–31]. The choice of RRT is a complex and time-pressured serious decision for patients. Studies have found that when faced with these kinds of decisions, as in our study, patients tend to use heuristic or intuitive decision-making strategies, rather than more systematic strategies where information is used to weigh benefits and risks [32].

We also found that many patients characterised treatment decision-making as very difficult or impossible. This could be explained by previous studies that found that patients may feel too ill in the pre-dialysis period to make a decision [8], possibly reflecting reduced cognitive functioning associated with reducing kidney function [14, 27]. Our patients’ reports of distress or trauma in the transition to RRT, whilst not new and reported in previous studies [27, 30, 33], may also help to explain these reported difficulties with decision-making. Cancer studies have found that emotional distress can impede patients’ understanding of information [34, 35], whilst the process of PDE itself may also contribute by adding emotional distress [7]. In addition to this aspect of patient experience, our finding that patients valued opportunities to talk to other patients about their treatment experiences and did this informally, is in line with recent studies [25].

Taken together, these findings suggest that PDE undertaken in the pre-dialysis period may not be effective for some patients, and the timing of PDE may need to extend beyond the pre-dialysis period. This may be appropriate for patients who are highly distressed in the pre-dialysis period, or patients who become open to other treatments only once they have themselves started treatment. Although the continuation of education beyond the pre-dialysis period is also supported by systematic review evidence [21] and clinical guidelines [15, 16], this would be a significant change in practice, as none of the study sites were providing on-going education or undertook formal treatment reviews as part of the RRT pathway.

## Conclusions

Our findings have highlighted a number of important issues for PDE. The finding that patients want improvements to teaching methods and materials is not new, and demonstrates that PDE may still have some way to go in meeting patients’ expectations, despite these issues having been highlighted for 10 years or more. This would involve specialist staff having access to a more diverse range of educational materials and using teaching methods which suit each patient’s learning style. Whilst these improvements would be relatively easy to implement, we also conclude that the approach to PDE needs to change. A much more individualised approach is required which takes account of the wide variation in patients’ motivation and interest in making treatment choices. Staff would need to help patients apply information to their own lives, taking account of living circumstances, values and priorities, and consider how psychosocial barriers to preferred treatments might be overcome. This is more akin to counselling than education and would demand a higher level of skill and training for specialist PDE staff. In addition to these improvements to formal PDE, we also conclude that renal units need to recognise that informal education takes place through casual conversations between staff and patients. We therefore recommend that *all* renal staff should be trained about all treatment options, irrespective of their role in PDE, so that they are more in tune with the complexities and difficulties patients face when considering treatment options. All staff would then also be to handle patients’ informal queries in an informed and unbiased way.

Even if the above improvements are made to PDE, we conclude that significant proportions of patients will still not benefit from it. If in the pre-dialysis period, significant numbers of patients find treatment information too complex to process, find decision-making difficult, feel too ill or too distressed to make decisions, and if some patients become more open to some treatment options only once they are on RRT, then education must continue into the RRT treatment phase as a routine part of the pathway. We also suggest that decisions made in the pre-dialysis period may not be optimal for significant numbers of patients and should therefore be considered temporary, with reviews built into the pathway so that there are structured opportunities for patients to revisit their treatment choices. We conclude that the phrase ‘PDE’ is a misnomer and argue instead for referring to on-going RRT education which starts in the pre-dialysis period and continues through into dialysis treatment.

Finally, we argue for the provision of emotional support both pre-dialysis and in the first year, once RRT has begun. This could be incorporated into education, which would also need to take account of psychosocial barriers to treatment and coping strategies. This could help patients to make decisions that are best for them in the medium-term rather than in response to the very real distress they may experience as they approach the transition to RRT.

### Strengths and limitations

The inclusion of four study sites, which varied in geographical location and patient demography, was a strength. The relatively large interview sample sizes lend weight to the findings, alongside the purposive patient sampling, which captured diverse patient experiences. The main limitation is that we did not set out to study PDE as a stand-alone topic. Had we done so, a mixed methods study would have been preferable, so that we could explore findings qualitatively and quantitatively. Another limitation is that sites may not be typical because they were working towards a target for home dialysis uptake. This had led to scrutiny of all aspects of the pathway, including PDE, and it might therefore be expected that PDE was more advanced in these sites compared with the rest of the country. However, the finding that improvements to PDE were still required suggests that there are enduring issues which are likely to be relevant to renal units elsewhere.

## Abbreviations

CKD stage 5: Chronic kidney disease stage 5
HD: Haemodialysis
HHD: Home haemodialysis
PD: Peritoneal dialysis
PDE: Pre-dialysis education
RRT: Renal replacement therapy
